# The *Trichoderma atroviride* Strains P1 and IMI 206040 Differ in Their Light-Response and VOC Production

**DOI:** 10.3390/molecules25010208

**Published:** 2020-01-03

**Authors:** Verena Speckbacher, Veronika Ruzsanyi, Modestus Wigger, Susanne Zeilinger

**Affiliations:** 1 Department of Microbiology, University of Innsbruck, 6020 Innsbruck, Austria; verena.speckbacher@uibk.ac.at; 2 Breath Research Institute, University of Innsbruck, 6850 Dornbirn, Austria; veronika.ruzsanyi@uibk.ac.at; 3 Umweltmonitoring und Forensische Chemie, Hochschule Hamm-Lippstadt, 59063 Hamm, Germany; modestus.wigger@stud.hshl.de

**Keywords:** *Trichoderma atroviride*, mycoparasitism, secondary metabolites, volatile organic compounds (VOCs), photoconidiation, fungi, 2-octanone, injury response, light response, *Fusarium oxysporum*

## Abstract

*Trichoderma atroviride* is a strong necrotrophic mycoparasite antagonizing and feeding on a broad range of fungal phytopathogens. It further beneficially acts on plants by enhancing growth in root and shoot and inducing systemic resistance. Volatile organic compounds (VOCs) are playing a major role in all those processes. Light is an important modulator of secondary metabolite biosynthesis, but its influence has often been neglected in research on fungal volatiles. To date, *T. atroviride* IMI 206040 and *T. atroviride* P1 are among the most frequently studied *T. atroviride* strains and hence are used as model organisms to study mycoparasitism and photoconidiation. However, there are no studies available, which systematically and comparatively analyzed putative differences between these strains regarding their light-dependent behavior and VOC biosynthesis. We therefore explored the influence of light on conidiation and the mycoparasitic interaction as well as the light-dependent production of VOCs in both strains. Our data show that in contrast to *T. atroviride* IMI 206040 conidiation in strain P1 is independent of light. Furthermore, significant strain- and light-dependent differences in the production of several VOCs between the two strains became evident, indicating that *T. atroviride* P1 could be a better candidate for plant protection than IMI 206040.

## 1. Introduction

Filamentous fungi are prolific producers of volatile organic compounds (VOCs), small carbon-based substances that readily enter the gas phase and that are derived from both, primary and secondary metabolism. Fungal VOCs are produced as mixtures of chemically diverse compounds, with the produced VOC profile being species- and even strain-specific and greatly depending on the developmental stage of the fungus as well as environmental factors [1,2,3]. They are involved in various biological processes including communication with other organisms such as plants and microbes as well as self-signaling. VOC emission by fungi serves in interaction with animals as reported for 1-octen-3-ol produced by the mushroom *Clitopilus prunulus* for deterring banana slugs and by the wood-rotting fungus *Trametes gibbosa* for attracting fungus-eating beetles [4,5]. Fungal VOCs further can impact plants by activating defense responses and affecting plant growth, as well as directly inhibiting the proliferation of phytopathogens [6].

Members of the fungal genus *Trichoderma* are efficient mycoparasites that antagonize a wide range of phytopathogenic fungi by direct parasitism employing secreted antifungal hydrolytic enzymes and metabolites [7]. At least 480 different VOCs have been identified from *Trichoderma* species yet, with 6-pentyl-2*H*-pyran-2-one (6PP) being one of the first volatiles isolated from this fungal genus [8,9]. *Trichoderma*-emitted VOCs have been connected to the antagonistic activity of these fungi, as they were shown to reduce the mycelial growth of fungal plant pathogens [8,10] as well as wood decay fungi [11]. However, only recently VOC production in co-cultivation was addressed, showing that interaction of *Trichoderma* spp. such as *T. harzianum, T. hamatum* and *T. velutinum* with the ectomycorrhizal fungus *Laccaria bicolor* dramatically altered the VOC emission patterns [12]. In addition to VOCs with bioactivity against fungi, *Trichoderma* spp. release volatiles that affect plant development and immunity. *Arabidopsis* plants exposed to the pool of VOCs emitted by *T. viride* showed increased lateral root formation and growth, and comparable results were obtained with *T. virens* and *T. pseudokoningii* volatiles [13,14,15]. Similarly, *T. atroviride*-derived 6PP stimulated tomato plant growth and systemic defense; however, the effects were concentration-dependent [8]. In addition to 6-PP, 24 members of the compound classes of alkanes, alcohols, ketones, lactones, furanes, monoterpenes, and sesquiterpenes—including typical fungal C8-compounds—as well as several volatiles not known to be produced by *Trichoderma* before, were identified in the headspace of cultures of *T. atroviride* strain P1 (ATCC 74058) [16]. C8-compounds such as 1-octen-3-ol, 3-octanol and 3-octanone are end-products of fatty acid metabolism [17] and act as signaling molecules regulating fungal development and inter-colony communication. In *T. atroviride*, these volatiles are increasingly produced by conidiating cultures and were described to act as elicitors of conidiation [18]. In addition, 1-octen-3-ol production by *T. atroviride* was up-regulated upon treatment of the fungus with the *Fusarium*-derived mycotoxin fusaric acid, while levels of other VOCs such as 6PP, alpha-phellandrene, and beta-phellandrene decreased [16].

Light, especially in the UV/blue spectrum, is an important environmental cue that triggers asexual reproduction in many fungal species [19]. In *Trichoderma,* most studies on photoconidiation have been performed with *T. atroviride* strain IMI 206040 as a model [20,21,22,23,24,25,26]. In complete darkness, *T. atroviride* IMI 206040 has been reported to grow infinitely as mycelium, while exposure to light induces the formation of green conidia [27,28].

In the present study, two different strains (*T. atroviride* P1, ATCC 74058 and *T. atroviride* IMI 206040) of the strong mycoparasite *T. atroviride* were analyzed for their differences in VOC biosynthesis by an in-house made high-resolution ion mobility spectrometer (IMS) with gas chromatographic (GC) pre-separation. Despite the fact that *T. atroviride* is a model to study photoconidiation, no studies have systematically and comparatively analyzed putative strain-, or light-dependent differences in the composition of VOC mixtures released by these fungi. We hence explored and compared their VOC profiles along a cultivation period of 120 h in complete darkness and upon exposure to light, as well as during the mycoparasitic interaction with the host fungi *Rhizoctonia solani* and *Fusarium oxysporum*. Remarkable differences not only in VOC production, but also in light-dependent conidiation and in the mycoparasitism on *F. oxysporum* became evident.

## 2. Results

### 2.1. The Vegetative Growth Rate of T. atroviride Is Strain- and Light- Dependent

**Figure 1:**
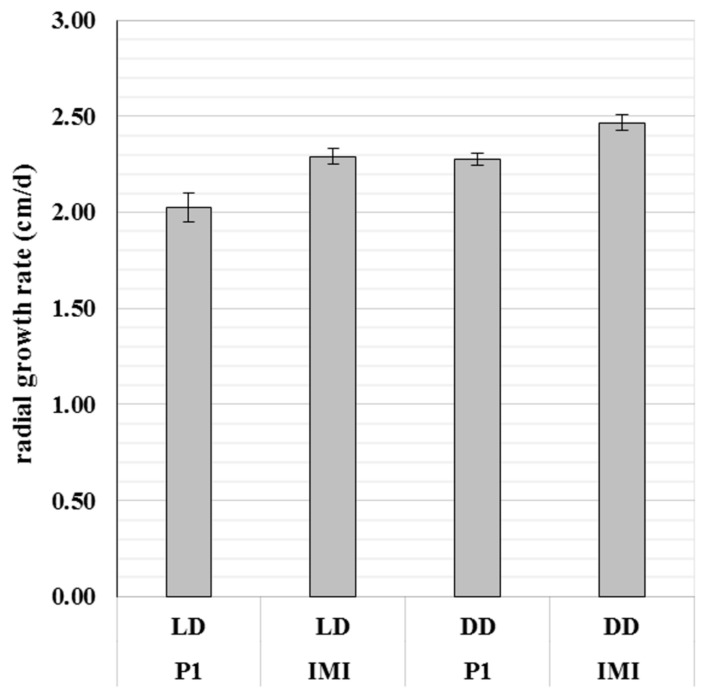
Strain-specific differences in radial growth of *Trichoderma atroviride*. The radial growth rate (cm/d) of *T. atroviride* P1 (P1) and IMI 206040 (IMI) after three days of cultivation on PDA at 25 °C under light-dark (LD) conditions or complete darkness (DD). Results shown are means ± SD (*n* = 4).

Upon cultivation on PDA plates, the radial growth rate differed between *T. atroviride* strains P1 and IMI 206040. *T. atroviride* IMI 206040 exhibited a higher radial growth rate than *T. atroviride* P1, irrespective of the applied light regime. However, both strains showed enhanced radial growth upon cultivation in complete darkness compared to light-dark conditions (Figure 1).

### 2.2. Asexual Sporulation in T. atroviride Is Strain- and Light-Dependent

**Figure 2:**
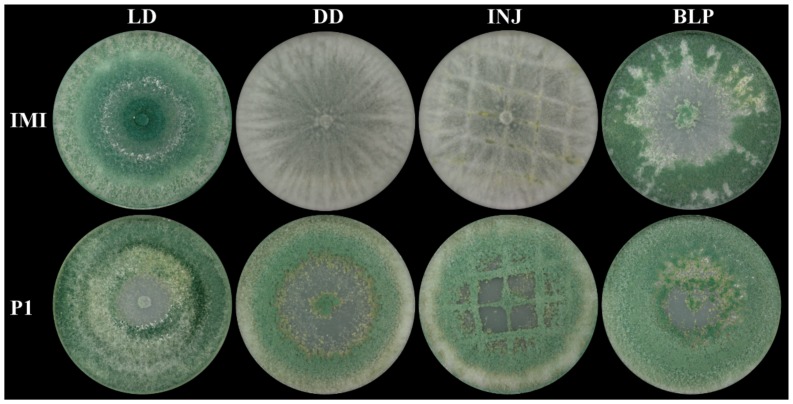
Strain-specific differences in conidiation upon growth under different light regimes and upon mechanical injury. *T. atroviride* P1 (P1) and *T. atroviride* IMI 206040 (IMI) were grown on PDA at 25 °C for five days under light-dark (LD) conditions or in complete darkness (DD). For induction of conidiogenesis, the fungi were grown in complete darkness for two days, treated by either mechanical injury (INJ) or a 10 min blue-light pulse (BLP) followed by incubation for further three days in complete darkness. A representative image of four biological replicates (*n* = 4) is shown.

Comparative analysis of *T. atroviride* P1 and IMI 206040 under conidiation-inducing conditions revealed significant differences between the two strains. In *T. atroviride* IMI 206040, asexual sporulation only occurred under light-dark conditions, while conidia were not formed upon cultivation in complete darkness. According to previous reports [29], conidiation could further be triggered in dark-grown *T. atroviride* IMI 206040 by mechanical injury or a pulse of blue light, respectively. In strain IMI 206040 injury resulted in low conidiation along the cutting sites only, whereas blue light treatment led to the production of massive amounts of heavily pigmented conidia. In contrast, *T. atroviride* P1 fully conidiated even upon growth in complete darkness. Mechanical injury led to strong conidiation and the generation of scarring tissue along the cutting sites in this strain (Figure 2).

### 2.3. The Mycoparasitic Activity of T. atroviride Is Strain- and Light-Dependent

**Figure 3:**
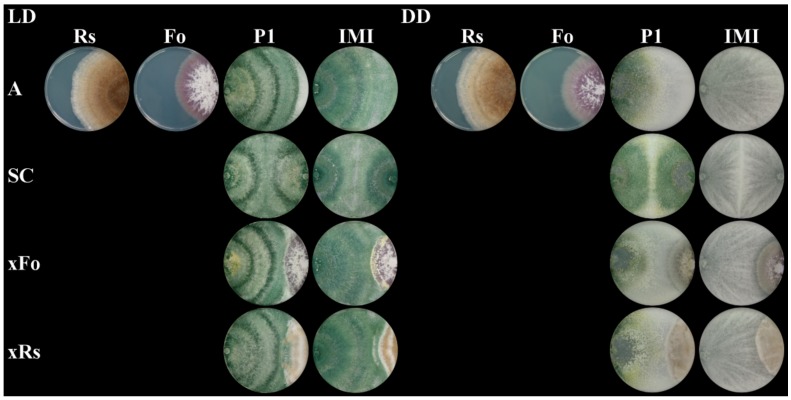
Strain-specific differences in the mycoparasitic behavior against *Fusarium oxysporum* and *Rhizoctonia solani* upon incubation under different light regimes. Plate confrontation assays of the mycoparasites *T. atroviride* P1 (P1) and *T. atroviride* IMI 206040 (IMI) against the host fungi *F. oxysporum* (Fo) and *R. solani* (Rs). As controls, all strains were grown alone (A). The mycoparasites were as well grown in self confrontation (SC) and in direct confrontation with *F. oxysporum* (xFo) or *R. solani* (xRs) on PDA at 25 °C for five days under either light-dark (LD) conditions or complete darkness (DD). A representative image of four biological replicates (*n* = 4) is shown.

In direct confrontation assays, the antagonistic activities against the tested host fungi differed between the two *T. atroviride* strains and turned out to be influenced by light. Upon growth in light–dark conditions, neither *T. atroviride* IMI 206040 nor strain P1 were able to fully overgrow and mycoparasitize the two tested host fungi *R. solani* and *F. oxysporum*. However, maybe due to its higher growth rate, *T. atroviride* IMI 206040 made better progress in overgrowing both hosts compared to *T. atroviride* P1. Upon growth in complete darkness both *T. atroviride* strains showed significantly increased antagonistic activities compared to light-dark conditions; they were able to fully overgrow and lyse *R. solani* within five days. Interestingly, *F. oxysporum* could only be fully overgrown and mycoparasitized by *T. atroviride* P1, which was even conidiating under these conditions, while *T. atroviride* IMI 206040 was less active against this host fungus and did not conidiate in darkness. These data suggest that upon growth in complete darkness, *T. atroviride* P1 is a stronger mycoparasite of *F. oxysporum* than *T. atroviride* IMI 206040 (Figure 3).

### 2.4. VOC Biosynthesis Is Mostly Strain-Specific but Also Light- and Host Dependent

VOC analyses of *T. atroviride* P1 and IMI 206040 along a period of 120 h upon axenic cultivation under light–dark conditions or in complete darkness as well as upon interaction with host fungi revealed more than 50 peaks in the GC–IMS chromatograms, which corresponded to approx. 20 to 25 VOCs. Since for IMS no commercial database, such as the NIST database for GC–MS, is available, we had to measure VOC standards and confirm the detected substances based on the characteristic IMS and GC data (drift and retention times, respectively). This resulted in the identification of a total of ten volatiles (Table 1). Except 2-octanone, which was solely detected in direct confrontation between *T. atroviride* and *F. oxysporum*, and 3-octanone, which was released by strain IMI 206040 solely upon light–dark conditions, all of the identified VOCs were produced by both strains irrespective of the cultivation condition. However, their amounts and course of release strongly differed between the different experimental setups. The PDA media itself did not emit any of the mentioned VOCs in significant concentrations.

#### 2.4.1. VOCs Emitted in Strain-Specific Amounts

Whereas 2-heptanone and ethanol were released in similar amounts to the headspace of the two tested *T. atroviride* strains, the amounts of 1-propanol, 2-methyl-propanol, 3-methylbutanal, 2-methyl-butanol, 3-methyl-1-butanol, 3-octanone and 1-octen-3-ol significantly varied between the two *T. atroviride* strains with strain P1 emitting considerably higher amounts than IMI 206040.

**Figure 4:**
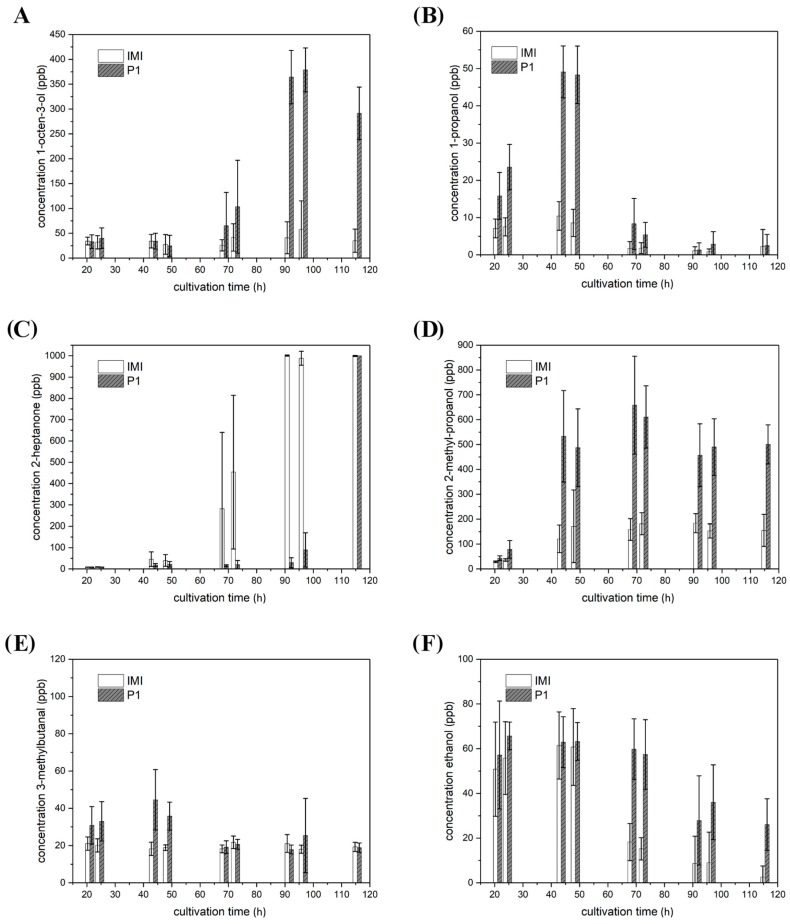
VOCs produced by *T. atroviride* P1 and IMI 206,040 in a strain-specific but light-independent manner. Levels (ppb) of (**A**) 1-octen 3-ol, (**B**) 1-propanol, (**C**) 2-heptanone, (**D**) 2-methyl-propanol, (**E**) 3-methylbutanal, and (**F**) ethanol detected in the headspace of *T. atroviride* P1 (P1) and *T. atroviride* IMI 206040 (IMI) cultures grown on PDA in Schott bottles. GC–IMS measurements were conducted along a cultivation period of 120 h. As the given volatiles were produced in similar amounts under light-dark conditions and in complete darkness, results shown are means ± SD (*n* = 8).

In *T. atroviride* P1, the release of 1-octen 3-ol to the headspace strongly increased from 68.5 h on and reached a maximal concentration of approx. 375 parts per billion (ppb) after 96.5 h of cultivation. In *T. atroviride* IMI 206040, 1-octen-3-ol emission peaked at the same time point with a maximal concentration of approx. 60 ppb meaning an 84% reduction compared to strain P1 (Figure 4A). The emission of 1-propanol increased during the early cultivation period, peaked at 43.5 h with approx. 50 ppb in *T. atroviride* P1 and afterwards decreased again. In *T. atroviride* IMI 206040, 1-propanol levels peaked at the same time point, but with a maximal concentration of approx. 10 ppb, which is equivalent to an 80% reduction compared to strain P1 (Figure 4B). 2-heptanone was increasingly released to the headspace over time, reaching and staying at a maximum of ≥1000 ppb at the end of the cultivation period in both *T. atroviride* strains. However, this volatile was already produced in considerable amounts of approx. 275 ppb after 68.5 h of cultivation in *T. atroviride* IMI 206040, while 2-heptanone emission by strain P1 was delayed and increased not before 96.5 h (Figure 4C). 2-methyl-propanol emission followed a similar time course in both *T. atroviride* strains. However, after a steep increase in the early phase of the cultivation, *T. atroviride* P1 produced maximal levels of approx. 650 ppb at the 68.5 h time-point, whereas *T. atroviride* IMI 206040 cultures only emitted up to approx. 175 ppb, which is equivalent to a 73% reduction compared to strain P1 (Figure 4D). 3-methyl-butanal emission by *T. atroviride* P1 reached 45 ppb after 43.5 h and afterwards decreased to 20 ppb until the end of cultivation, whereas in *T. atroviride* IMI 206040 approx. 20 ppb of this volatile were constantly emitted along the whole cultivation period (Figure 4E). The release of ethanol to the headspace increased at the early phases of the cultivation period, peaked at 24.5 h with approx. 65 ppb in *T. atroviride* P1 and afterwards decreased again to approx. 25 ppb. Young *T. atroviride* IMI 206040 cultures produced similar amounts of this volatile with a maximal level of approx. 60 ppb after 43.5 h of cultivation, but with a successive steeper decrease down to approx. 5 ppb at the end of the cultivation period (Figure 4F).

#### 2.4.2. VOCs Emitted in Strain-Specific Amounts in a Light-Dependent Manner

3-octanone, 2-methyl-butanol, and 3-methyl-1-butanol were not only secreted in a strain- but also in a light-dependent manner.

**Figure 5:**
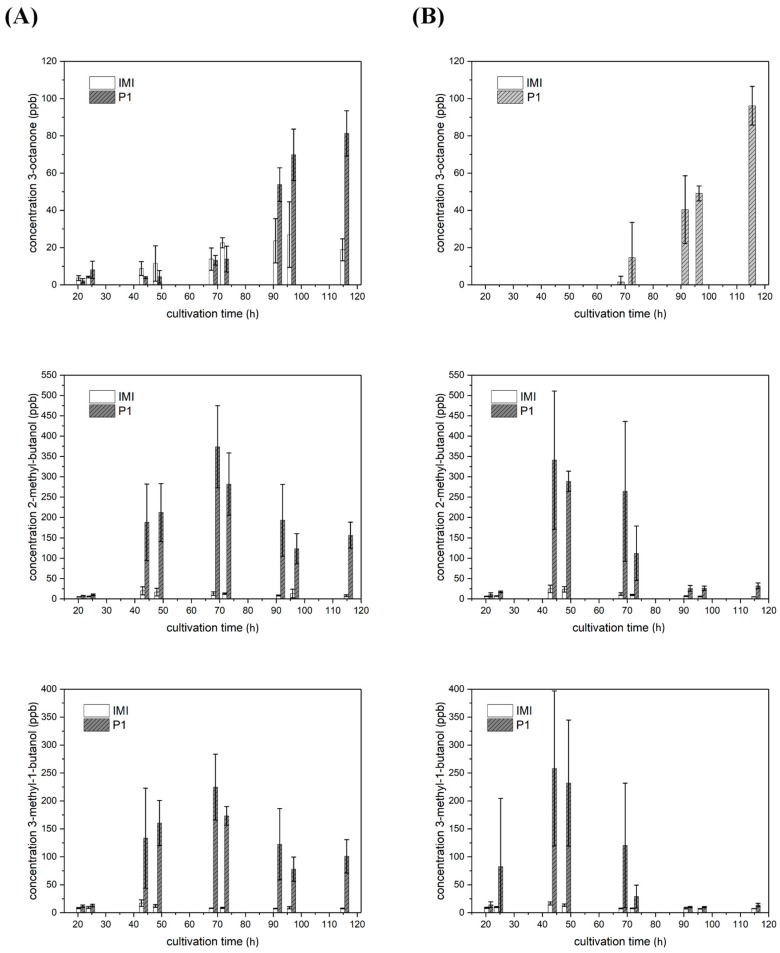
VOCs produced by *T. atroviride* P1 and IMI 206,040 in a strain-specific and light-dependent manner. Levels (ppb) of 3-octanone (row 1), 2-methyl-butanol (row 2), and 3-methyl-1-butanol (row 3) detected in the headspace of *T. atroviride* P1 (P1) and *T. atroviride* IMI 206040 (IMI) cultures grown on PDA in Schott bottles at 25 °C under (**A**) light-dark conditions or (**B**) in complete darkness. GC–IMS measurements were conducted along a cultivation period of 120 h. Results shown are means ± SD (*n* = 4).

Upon growth in complete darkness, *T. atroviride* IMI 206040 did not emit any 3-octanone. However, up to 25 ppb were measured after 96.5 h of cultivation under light-dark conditions. As the strain already conidiated at this time point in the presence of light, 3-octanone production could not only be light-induced but also conidiation-associated in *T. atroviride* IMI 206040. In contrast, 3-octanone emission by *T. atroviride* P1 was, similar to conidiation, light-independent and increased with cultivation time to a maximum of 80–95 ppb after 115.5 h of growth (Figure 5; row 1).

2-methyl-butanol and 3-methyl-1-butanol were produced light-independently by *T. atroviride* IMI 206040. Low amounts of approx. 25 ppb of both volatiles were detected under both light-dark conditions and complete darkness along the whole cultivation period of this strain (Figure 5 rows 2 and 3). In contrast, *T. atroviride* P1 emitted 2-methyl-butanol and 3-methyl-1-butanol in a slightly light-dependent manner. Upon cultivation under light-dark conditions, increasing amounts of both substances were released to the headspace with a peak of approx. 375 ppb of 2-methyl-butanol and approx. 225 ppb of 3-methyl-1-butanol after 68.5 h of cultivation. Similar maximal amounts were emitted by strain P1 upon growth in complete darkness; however, production and decline started earlier compared to light–dark conditions (Figure 5; rows 2 and 3).

#### 2.4.3. VOC Emission in Co-Culture

To assess the influence of a host fungus on the emission of VOCs by *T. atroviride*, both strains—IMI 206040 and P1—were co-cultured with *R. solani* or *F. oxysporum*.

**Figure 6:**
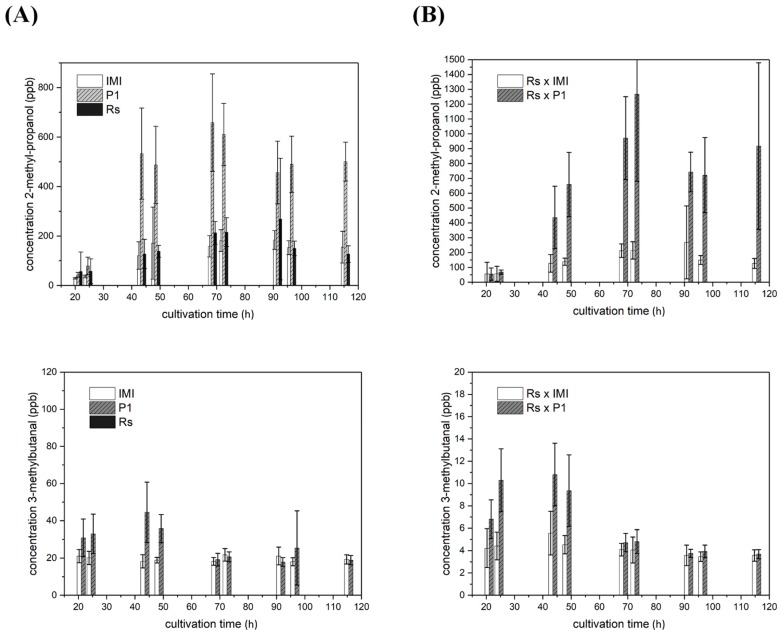
Emission levels of light-independently produced VOCs upon co-cultivation of *T. atroviride* P1 or IMI 206040 with *R. solani*. Levels (ppb) of 2-methyl-propanol (row 1) and 3-methylbutanal (row **2**) detected in the headspace of (**A**) axenic cultures of *T. atroviride* P1 (P1), *T. atroviride* IMI 206040 (IMI) and *R. solani* (Rs) or of (**B**) co-cultures of *T. atroviride* P1 or IMI 206040 confronted with *R. solani* (Rs × P1; Rs × IMI) upon cultivation on PDA in Schott bottles at 25 °C. GC–IMS measurements were conducted along a cultivation period of 120 h. As the given volatiles were produced in similar amounts under light-dark conditions and in complete darkness, results shown are means ± SD (*n* = 4).

Compared to axenic cultures of both *T. atroviride* strains and the fungal host, the amounts of 2-methyl-propanol and 3-methylbutanal strongly differed in co-cultures with *R. solani*. The host released a maximal amount of approx. 260 ppb 2-methyl-propanol after 91.5 h in a light-independent way, whereas it did not produce 3-methylbutanal upon axenic cultivation. The course of 2-methyl-propanol and 3-methylbutanal emission upon confrontation of *T. atroviride* with *R. solani* was similar to the axenic *T. atroviride* cultures. However, 2-methyl-propanol levels were enhanced in co-cultures involving *T. atroviride* P1 compared to the respective axenic cultures (Figure 6; row 1). In contrast, decreased amounts of 3-methylbutanal were released to the headspace by both *T. atroviride* strains upon confrontation with *R. solani* compared to axenic cultures (Figure 6; row 2).

Upon co-cultivation of *T. atroviride* with *F. oxysporum*, 1-propanol, 2-heptanone, and 3-octanone were produced in different amounts compared to the respective axenic cultures. In addition, 2-octanone was exclusively detected upon co-cultivation.

**Figure 7:**
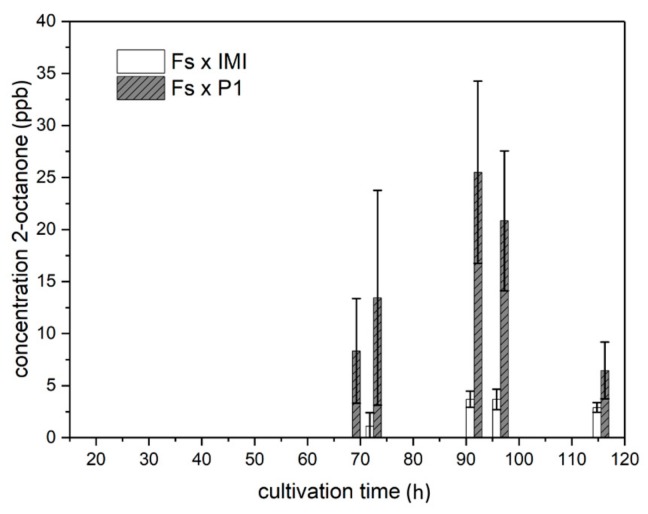
Exclusive, light-independent production of 2-octanone in direct confrontation of *T. atroviride* P1 and IMI 206040 with *F. oxysporum*. Levels (ppb) of 2-octanone detected in the headspace of co-cultures of *T. atroviride* P1 or IMI 206040 with *F. oxysporum* (Fo × P1; Fo × IMI) grown on PDA in Schott bottles at 25 °C. GC–IMS measurements were conducted along a cultivation period of 120 h. As 2-octanone emission in co-culture reached similar levels under light-dark conditions and in complete darkness, results shown are means ± SD (*n* = 4).

**Figure 8:**
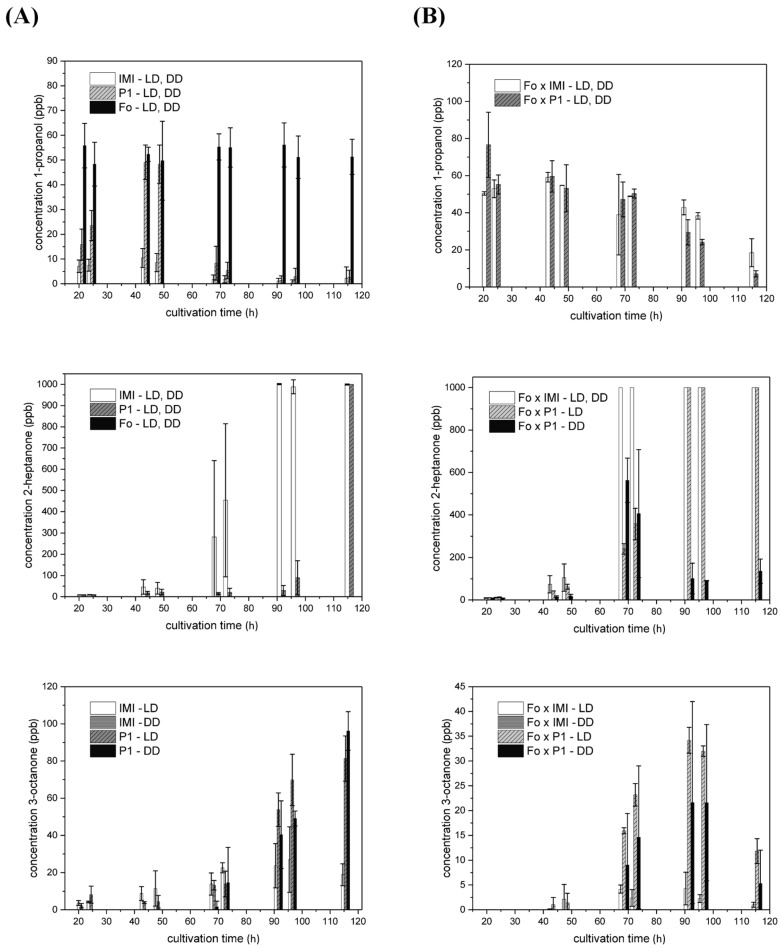
VOC production by *T. atroviride* P1 and IMI 206,040 upon interaction with *F. oxysporum*. Levels (ppb) of 1-propanol (row 1), 2-heptanone (row 2), and 3-octanone (row 3) detected in the headspace of (**A**) axenic cultures of *T. atroviride* P1 (P1), *T. atroviride* IMI 206040 (IMI), or *F. oxysporum* (Fo) or of (**B**) co-cultures of *T. atroviride* P1 or IMI 206040 with *F. oxysporum* (Fo × P1; Fo × IMI) on PDA in Schott bottles at 25 °C. Cultures were incubated either under light-dark conditions (LD) or in complete darkness (DD) as indicated. GC–IMS measurements were conducted along a cultivation period of 120 h. Results shown are means ± SD (*n* ≥ 3).

When grown in axenic culture, *F. oxysporum* constantly released approx. 55 ppb 1-propanol in a light-independent manner, whereas 2-heptanone, 3-octanone, and 2-octanone could not be detected under these conditions. However, confrontations of both *T. atroviride* strains with *F. oxysporum* led to the release of 2-octanone, which was not produced under any other condition tested in this study. Interestingly, 2-octanone emission during co-cultivation of strain P1 with *F. oxysporum* was significantly higher compared to the IMI 206040—*F. oxysporum* pairing. 2-octanone release started after 68.5 h of growth, peaked after 91.5 h with approx. 25 ppb in the co-culture involving strain P1 and with 4 ppb in the co-culture with strain IMI 206040 and afterwards decreased again (Figure 7). 1-propanol levels were increased, especially between 68.5 and 96.5 h of growth upon co-cultivation of *T. atroviride* P1 or IMI 206040 and *F. oxysporum* compared to the respective axenic cultures. In contrast to axenic cultures, which peaked between 40 and 50 h of cultivation, all co-cultivations with *F. oxysporum* resulted in high initial levels, which then constantly decreased over the cultivation period. (Figure 8; row 1).

Interestingly, 2-heptanone emission by *T. atroviride* P1 in co-culture with *F. oxysporum* was light-dependent. In dark-grown co-cultures, 2-heptanone production by strain P1 followed a completely different course compared to co-cultivation under light–dark conditions, IMI 206040—*F. oxysporum* pairings, and axenic cultivations. 2-heptanone levels peaked with approx. 550 ppb after 68.5 h of co-cultivation in darkness and afterwards rapidly declined again. In contrast, 2-heptanone concentrations in the headspace of co-cultures grown under light–dark conditions increased steadier and earlier than upon axenic growth and finally reached and stayed at ≥1000 ppb (Figure 8; row 2). A similar trend was evident in the co-culture of strain IMI 206040 and *F. oxysporum*.

Similar to axenic cultivation, 3-octanone was solely produced in the presence of light by strain IMI 206040, irrespective of the presence of *F. oxysporum*. In axenic cultures, 3-octanone release increased over the whole cultivation period. In contrast, co-cultivation with *F. oxysporum* resulted in maximal 3-octanone production after 91.5 h, although 3-octanone levels were about 50% reduced compared to axenic cultivation. A massive reduction in 3-octanone emission upon interaction with *F. oxysporum* was also evident in strain P1, especially upon cultivation in darkness. However, 3-octanone production in co-culture was triggered by light as strain P1 produced nearly the double amount of this volatile in co-culture with *F. oxysporum* under light-dark conditions compared to constant darkness (Figure 8; row 3).

## 3. Discussion

*Trichoderma atroviride’s* natural habitat is soil and soil itself is penetrated by light in its upmost layer (with the exception of far-red light) solely. From the ecological point of view, the observed enhanced radial growth of *T. atroviride* colonies upon incubation in complete darkness indicates that dark conditions were closer to the conditions prevailing in the natural habitat than direct exposure to light. In accordance with this assumption, both *T. atroviride* strains exhibited a higher ability of antagonizing *R. solani* and particularly *F. oxysporum* in darkness compared to light–dark conditions, under which the latter fungal host could not be overgrown. The faster growth of *T. atroviride* IMI 206040 compared to strain P1 could account for its higher mycoparasitic activity in light. On the other hand, the observed higher antagonistic potential of *T. atroviride* P1 against *F. oxysporum* under complete darkness indicates that this strain is a stronger mycoparasite of *Fusarium* than *T. atroviride* IMI 206040, especially since its radial growth rate was lower. Our results point out that the frequently applied light–dark cycle, exposing the fungi for 12 h to illumination with white light, is an artificial and stressful condition that results in reduced growth and reduced mycoparasitic activity. Since under natural conditions the contact with light usually is accompanied by higher temperatures, drought and exposure to mutagenic ultraviolet light, paralleled by an extensive onset of reactive oxygen species production [29,30], the observed light-triggered growth reduction seems plausible. A strategy to react to and protect from light is conidiation, which was reported to be induced by (a pulse of blue-) light as well as mechanical injury in *T. atroviride* IMI 206040 [31]. Surprisingly, we found that *T. atroviride* P1 similarly produces asexual spores in the presence as well as absence of light, which is in massive contrast to the lack of sporulation in darkness in strain IMI 206040. Differences between the two tested *T. atroviride* strains were also observed regarding their response to mycelial injury. While injury led to the formation of strongly conidiating hypertrophic scarring tissue along the cutting sites in strain P1, the injury response of strain IMI 206040 resulted in only slight conidiation along the cutting lines without an obvious formation of scarring tissue. In combination with its high resistance against several fungicides [32], the light-independent conidiation behavior of *T. atroviride* P1 could be a major advantage for its application as a biocontrol agent in the field, since conidia, which are the main form of application, can be produced in a straightforward manner. Furthermore, belowground conidiation could also be a major advantage for long-term persistence of the mycoparasite in soil.

VOCs are playing important and versatile ecological roles in various intra- and inter-species or even inter-kingdom interactions [2,6]. A well-known example of such “multi-purpose” fungal VOCs are the eight-carbon volatiles 1-octene-3-ol and 3-octanone, which are contributing to the characteristic mushroom aroma and therefore are applied as food odorants in industry [33]. Furthermore, 1-octen-3-ol is known to act as mosquito attractant [34] and both eight-carbon volatiles were found to endogenously regulate conidial germination as well as the induction and inhibition of conidiation in a concentration-dependent manner [18,35,36,37]. Since *T. atroviride* IMI 206040 does not conidiate under dark conditions, the complete absence of 3-octanone in all samples obtained from dark-grown cultures of this strain indicates that 3-octanone is exclusively produced by conidia or under conidiation-inducing conditions. The substantially higher release of the majority of VOCs detected in this study by *T. atroviride* P1 suggests that this strain has a higher potential for biotechnological VOC production than strain IMI 206040. As described above, the versatile ecological roles of several fungal VOCs are well known; however, the regulation of their production along cultivation, like in dependence of culture age, and in response to stress is sparsely understood. In plants, VOC biosynthesis is regulated by abiotic stress, in particular by light and temperature stress, as well as biotic stress [38,39]. Similar to plants, fungi are relatively immobile and a regulation of VOC production as a stress response or coping strategy therefore is very likely. In this study, we found the three VOCs 3-octanone, 2-methyl-butanol, and 3-methyl-1-butanol to be released by *T. atroviride* axenic cultures in a light-dependent manner. Since the natural habitat of *T. atroviride* is soil, light and in particular UV irradiation is a stressful condition leading to the release of reactive oxygen species and to asexual reproduction [28,29]. In that respect, the light-dependent emission of 3-octanone, 2-methyl-butanol, and 3-methyl-1-butanol could play a role in the abiotic stress response to light in *T. atroviride*. Interestingly, 2-heptanone production by *T. atroviride* P1 during co-culture with *F. oxysporum* was also regulated in a light-dependent manner. Compared to *R. solani*, *F. oxysporum* is a more challenging host fungus for *T. atroviride*, which in our study could only be fully overgrown by strain P1 under dark conditions. In contrast, strain IMI 206040 was unable to fully parasitize *F. oxysporum*, irrespective of the applied light regime. In dark-grown co-cultures of *T. atroviride* P1 and *F. oxysporum*, only low 2-heptanone levels were emitted, which peaked and then rapidly decreased again. In contrast, confrontation of strain P1 with *F. oxysporum* in the presence of light as well as IMI 206040—*F. oxysporum* co-cultures and axenic cultivation of both *T. atroviride* strains in darkness and light resulted in a constant increase of 2-heptanone emission reaching and staying at levels of ≥1000 ppb. Low 2-heptanone levels hence paralleled the successful parasitism of *F. oxysporum* by *T. atroviride* P1, while the light-triggered emission of high levels of this volatile was associated with difficulties of *T. atroviride* to overgrow and lyse this host. *F. oxysporum* spp. are known to produce a plethora of toxic secondary metabolites [40,41], of which e.g., fumonisin is increasingly produced in the presence of light [42]. 2-hepanone emission by *T. atroviride* could be part of a coping strategy against abiotic (light) and biotic (*F. oxysporum* mycotoxins) stress.

*F. oxysporum*—*T. atroviride* co-cultivation further resulted in the production of 2-octanone, a volatile that was not emitted by any of the interaction partners upon axenic growth. Interestingly, a recent study revealed that deletion of the histone deacetylase-encoding gene *hda-2* in *T. atroviride* IMI 206040 leads to the biosynthesis of 2-octanone as a new volatile in this strain [43]. This report together with our results points out that 2-octanone biosynthesis genes could be shut-down by a repressive chromatin structure and could be activated epigenetically as a consequence of environmental stimuli, in this case the interaction with *F. oxysporum*. Accordingly, the bacterium *Streptomyces rapamycinicus* triggered the expression of the silent gene cluster responsible for orsellinic acid production in *Aspergillus nidulans* by activation of the histone acetyltransferase GcnE [44].

In summary, our study revealed significant differences between the two tested *T. atroviride* strains regarding injury-response, light-dependent regulation of conidiation and mycoparasitic activity as well as VOC production. Besides volatiles with strain- and light-dependent biosynthesis patterns, we found an interaction-dependent regulation of the biosynthesis of individual substances and obtained evidence, that the biosynthesis of certain VOCs is specifically linked to abiotic and/or biotic stress responses in *T. atroviride*. Additional effort should be undertaken to further decipher the multifactorial regulation of secondary metabolite biosynthesis in *Trichoderma* mycoparasites.

## 4. Materials and Methods

### 4.1. Fungal Strains and Culture Conditions

*Trichoderma atroviride* P1 (ATCC 74058), *T. atroviride* IMI 206040, *Fusarium oxysporum* f. sp. *lycopersici* strain 4287 and *Rhizoctonia solani* strain AG-5 were used in this study.

Pre-cultivation was done by passaging a 6 mm diameter agar plug of the actively growing colony margin at least two times after 1.5 days each upside down to the centre of a fresh petri dish (94 mm × 16 mm, Greiner Bio-One GmbH, Kremsmünster, Austria) containing 25 mL potato dextrose agar (PDA; Becton, Dickinson and Company, Le Pont De Claix, France). Plates were incubated at 25 °C under light-dark conditions (12:12 h, 300 Lux; Snijders Micro Clima-Series TM Labs Economic Lux Chamber; Snijders Labs, Tiburg, The Netherlands) or under complete darkness.

For determination of the radial growth rate of the two *T. atroviride* strains, 6 mm diameter agar plugs of the actively growing colony margins from the pre-cultures were inoculated in quadruplicates at the outmost margin of fresh agar plates. Plates were incubated at 25 °C under light-dark conditions (12:12 h; 300 Lux) or under complete darkness for three days. The radial growth rate was measured and calculated (cm/d) after three days of growth.

### 4.2. Mechanical Injury- and Blue-Light-Induced Conidiation Assays

Mechanical injury- and blue-light-induced conidiation assays were performed in quadruplicates according to [31] with slight modifications. Six millimeter diameter agar plugs of the actively growing colony margins from the pre-cultures were inoculated upside-down at the centre of fresh agar plates. Cultures were incubated at 25 °C for five days under light-dark conditions or under complete darkness. After two days of incubation in complete darkness, conidiation was induced by mycelial injury, which was set by cutting five horizontal and five vertical lines with a scalpel under red safety light. Alternatively, a 10 min pulse of blue light was set with a blue light source (Blacklight Blue UV-A Lamp: Supratec L18 W/73, 300–400 nm; 25 Lux; Osram GmbH, Garching, Germany; distance 19 cm). Plates were further incubated under complete darkness for a total of five days. Photos were taken after five days of growth.

### 4.3. Dual Plate Confrontation Assays

Dual plate confrontation assays were performed in quadruplicates according to [30] with slight modifications. Six millimeter diameter agar plugs of the actively growing colony margins from the pre-cultures of *T. atroviride* and a respective host fungus were inoculated on opposite sides at the outmost margin of fresh agar plates. As controls, all strains were additionally grown alone; the mycoparasites were also grown in self-confrontations. Due to its slower radial growth rate, *F. oxysporum* was inoculated 24 h earlier than the other strains. Fungi were grown at 25 °C under light-dark conditions or under complete darkness for five and six days, respectively. Pictures were taken at the end of the cultivation period to document the progress of the mycoparasitic attack.

### 4.4. Gas Chromatography–Ion Mobility Spectrometry (GC–IMS) Analysis of Headspace VOCs

For gas chromatography–ion mobility spectrometry (GC–IMS) analysis, a 6 mm diameter agar plug of the actively growing colony margin from the pre-culture was inoculated upside-down at the outmost margin of a 150 mL glass bottle (Duran GmbH, Mainz, Germany) filled with 25 mL PDA at the bottom. Fungi were grown in axenic culture or direct confrontation in the glass bottles according to the description above. As a control, PDA was also measured alone. The bottles were closed with Teflon^®^ screw caps (Bohlender^TM^, Merck, Vienna, Austria) with two openings for air in- and outlet. For dark conditions, the bottles were covered with several layers of aluminum foil. Due to a slower radial growth rate, *F. oxysporum* was pre-grown for 24 h. The two *T. atroviride* strains and *R. solani* were pre-grown for 20 h at 25 °C under light-dark conditions (12:12 h; 300 Lux) or under complete darkness before flasks were connected to the MS device.

For headspace measurements, cultures in glass bottles were held in an incubator and connected in a gastight way, parallel to each other. To avoid condensation, the inner temperature of the oven (so the temperature of the headspace air) was held at 40 °C, while the water bath was kept at 23 ± 2 °C. For ventilation, purified air with 5 mL/min flow was continuously streaming through the flasks and regulated by four mass flow controllers (MCF, Bronkhorst, Ruurlo, The Netherlands). Additionally, 5 mL/min purified air was connected as dilution flow to the flasks to reduce moisture in the samples. Headspace air samples were collected in the incubator at 40 °C in 100 mL and 250 mL glass syringes (Socorex, Ecublens, Switzerland). Sampling and measurement were performed 21, 24.5, 43.5, 48.5, 68.5, 72.5, 91.5, 96.5 and 115.5 h after inoculation.

A high resolution GC–IMS developed at Leibniz Universität, Hannover was applied to monitor the emitted VOCs. Samples were injected immediately after collection through a heated inlet (40 °C) into the GC column using a stainless steel sample loop (200 µL) installed on a six-way valve (VICI AG International, Schenkon, Switzerland). Volatiles were separated using a RTX volatiles column (10 m × 0.53 mm × 2 µm, Restek GmbH, Bad Homburg, Germany) working at a constant temperature of 50 °C. The carrier-gas-flow-rate program was as follows: 3 mL/min for 10 min and then 10 mL/min for another 10 min, resulting in a total GC-runtime of 20 min. The IMS, with a drift tube length of 7.5 cm, provided a resolving power of R = 90 using a drift voltage of 5 kV. The instrument operated at 40 °C with purified air as drift gas at the flow of 150 mL/min and 10 mbar above the ambient pressure. For ionization of the volatiles, a radioactive β-emitter ^3^H (300 MBq) was used. A detailed description of the system can be found in [31]. Chromatographic data was acquired using the Agilent Chemstation Software (GC-MS Data Analysis from Agilent, Waldbronn, Germany). Data were analyzed using the software OpenChrom (vers. 1.4.0, Lablicate GmbH, Hamburg, Germany) and the mass spectrum library NIST 2008 (Gatesburg, PA, USA) was applied for identification.

## Figures and Tables

**Table 1:** Volatile organic compounds (VOCs) identified via gas chromatography–ion mobility spectrometry (GC–IMS) in the headspace of *T. atroviride* P1 and IMI 206040.

Formula	Name of Compound	CAS-Number
C_2_H_6_O	ethanol	64-17-5
C_3_H_8_O	1-propanol	71-23-8
C_4_H_10_O	2-methyl-propanol	78-83-1
C_5_H_10_O	3-methylbutanal	590-86-3
C_5_H_12_O	2-methyl-butanol	137-32-6
C_5_H_12_O	3-methyl-1-butanol	123-51-3
C_7_H_14_O	2-heptanone	110-43-0
C_8_H_16_O	1-octen-3-ol	3391-86-4
C_8_H_16_O	2-octanone	111-13-7
C_8_H_16_O	3-octanone	106-68-3
